# Toward a unified information framework for cell atlas assembly

**DOI:** 10.1093/nsr/nwab179

**Published:** 2021-09-27

**Authors:** Sijie Chen, Yanting Luo, Haoxiang Gao, Fanhong Li, Jiaqi Li, Yixin Chen, Renke You, Hairong Lv, Kui Hua, Rui Jiang, Xuegong Zhang

**Affiliations:** Bioinformatics Division of BNRIST and Department of Automation, MOE Key Lab of Bioinformatics, Tsinghua University, China; Bioinformatics Division of BNRIST and Department of Automation, MOE Key Lab of Bioinformatics, Tsinghua University, China; Bioinformatics Division of BNRIST and Department of Automation, MOE Key Lab of Bioinformatics, Tsinghua University, China; Bioinformatics Division of BNRIST and Department of Automation, MOE Key Lab of Bioinformatics, Tsinghua University, China; Bioinformatics Division of BNRIST and Department of Automation, MOE Key Lab of Bioinformatics, Tsinghua University, China; Bioinformatics Division of BNRIST and Department of Automation, MOE Key Lab of Bioinformatics, Tsinghua University, China; Fuzhou Institute of Data Technology, China; Bioinformatics Division of BNRIST and Department of Automation, MOE Key Lab of Bioinformatics, Tsinghua University, China; Fuzhou Institute of Data Technology, China; Bioinformatics Division of BNRIST and Department of Automation, MOE Key Lab of Bioinformatics, Tsinghua University, China; Bioinformatics Division of BNRIST and Department of Automation, MOE Key Lab of Bioinformatics, Tsinghua University, China; Bioinformatics Division of BNRIST and Department of Automation, MOE Key Lab of Bioinformatics, Tsinghua University, China; School of Medicine, Tsinghua University, China; School of Life Sciences, Center for Synthetic and Systems Biology, Tsinghua University, China

## Abstract

This perspective discusses the need and directions for the development of a unified information framework to enable the assembly of cell atlases and a revolution in medical research on the virtual body of assembled cell systems.

Cells are the basic structural and functional unit of the human body. Virtually all cells of a human body possess the same genome, but they exhibit colorful heterogeneities in phenotypes and functions. The heterogeneities include rich patterns and variabilities in the transcriptomic, epigenomic and proteomic features of the cells. Building an atlas of human cells with the biomolecular properties of all cell types in all organs is essential for understanding the human body, and will provide a fundamental reference for studies on human health and diseases. Its importance to science is regarded as comparable to, or greater than, that of the Human Genome Project [1,2]. With the boom in single-cell omics technologies, especially single-cell RNA-sequencing (scRNA-seq), scientists initiated ambitious programs such as the Human Cell Atlas (HCA) [1], the Human BioMolecular Atlas Program (HuBMAP) [2] and Human Developmental Cell Atlas (HDCA) [3] for building cell atlases. Meanwhile, rapid democratization of the technology is enabling labs all over the world to generate single-cell data more quickly than big consortiums. Assembling scattered data has become an alternative approach for building cell atlases.

Cell atlas assembly faces major informatics challenges. The challenges are intrinsic to the complexity of human cell systems, and apply to the assembly of both scattered data and data collected with coordination.

The first challenge is the size of data in both depth and width. The human genome has ${\sim}3 \times {10^9}$ nucleotides or bases. Sequence segments are mapped to this one-dimensional backbone in Human Genome Project (HGP). Cells are the ‘bases’ of a human cell atlas. The total number of cells of an adult human is estimated to be around ${10^{13\sim 14}}$. Each cell possesses a genome and a variety of gene expression products. Current scRNA-seq technologies measure the expression of protein-coding genes ($\sim\!\! {10^4}$) in cells. With the future inclusion of alternatively spliced transcripts, non-coding transcripts and epigenomic features such as methylation and chromatin openness measured by other technologies, the feature number for each cell could be >${10^6}$. The size of a data table to store information along the genome may not exceed ${\sim} {10^9} \times {10^{2\sim 3}}$. But for a complete human cell atlas, the size could eventually reach ${\sim} {10^{13\sim 14}} \times {10^{6\sim 7}}$. It is arguable that not all the data are indispensable, but it is too early to judge what data can be discarded. Even after removing redundancy, the data size of cell atlases will still be magnitudes larger than that of a genome both in width and depth, way beyond the capacity of conventional relational database technologies.

The second challenge is the lack of a unified index of cells. The human genome has a natural 1-D index by which all information is organized. Some scientists believe that the anatomic geometry is a natural index of human cells, and propose the development of a common coordinate framework (CCF) to align geometries between individuals [4]. This is no easy task, and to what extent such a CCF can work as the index of an atlas is questionable, since geometric locations of specific cells in organs are not deterministic or destined at the microscopic level. The ASCT+B table proposed by the HuBMAP consortium suggested the use of anatomic structures, cell types and biomarkers as indexes to all cells [5]. The complexity of the human body requires a multi-granular index system. The system should be expandable in its contents and volume as current knowledge on complex cell systems is still limited.

The third challenge is the lack of consistency in annotating cells. Cell types and cell states are two major concepts in cell atlas studies, but they are loosely defined in many contexts. Annotations are not always consistent across studies, and sometimes even within studies. The uncertainty and inconsistency further increase when dynamics in developmental or stimuli-response processes are considered. An expandable unified structured ontology in conjunction with the index system is needed to assemble data into atlases and make data reusable in the assembly.

An ideal cell atlas (Fig. 1) should assemble data of all organs into one unified repository rather than a collection of datasets. A unified information framework is essential. It should include an infrastructure capable of storing and retrieving all data of ideally unlimited size and dimensionality in the same repository, and an underlying information graph or network that can unify and effectively represent major knowledge and multifaceted annotations of the anatomic, cellular and functional properties of cells. The unified framework should provide indexes to cells with all properties, and enable convenient, efficient browsing, searching and reusing all or selected features of any cells and any subgroups seamlessly across data from different sources. There have been efforts to collect single-cell data and build integrative uses of the data [1,6–9].

**Figure 1.**
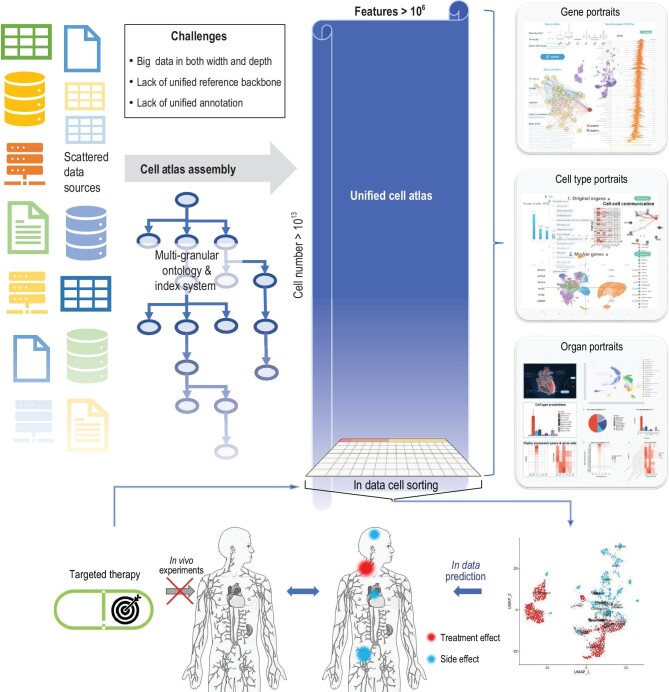
An ideal unified information framework for cell atlas assembly and applications. Many single-cell data are accumulated in the public domain. Assembling data from all sources provides a promising approach to building cell atlases, but there are major informatics challenges. A unified information framework is essential for cell atlas assembly. An expandable multi-granular ontology and index system is needed to unify uncoordinated data and assemble them into a giant data repository that is big in both depth and width. With sufficient data coverage and depth, a unified atlas will provide quantitative ‘holographic portraits’ of fundamental biological entities of the human body at anatomic, cellular and molecular levels. The unified framework will also provide revolutionary ways of conducting biological experiments such as using ‘in data’ cell sorting on the ‘virtual human body’ to identify off-target effects of targeted cancer therapy without doing *in vivo* experiments.

But a unified information framework is still lacking. Most large-scale bioinformatics databases, like those in NCBI, are organized in files indexed by their submitted order rather than intrinsic relations among data elements. A great advancement in people's handling and utilization of comprehensive data is the geographic information system (GIS), which captures, stores and displays all types of data relating to positions on the Earth's surface. A unified information framework for cell atlases should be like a super GIS that can encompass microscopic, mesoscopic and macroscopic data relating to cells in the human body, and a living GIS that can represent multifaceted functional data and relations beyond spatial maps.

To explore roadmaps for potential solutions, we developed a unified information framework, the *Ensemble Cell Atlas*, or ECA, as a prototype for a unified cell atlas to tackle these challenges [10]. It includes a wide-column *unified giant table* (uGT) to manage the big data, a *unified hierarchical annotation framework* (uHAF) for indexing and annotating cells at multiple granularities, and a search engine, ECAUGT (pronounced ‘e-caught’), to implement logic searching in the uGT. The framework allows cells to have unified indexes on all features that might be used as coordinates, including spatial and temporal features at multiple scales.

Using the framework, a primary version of the *human Ensemble Cell Atlas* (hECA) was built by assembling scattered scRNA-seq data covering 38 organs, with data of >1 million cells of >40 000 features. This coverage is shallow compared to the final goal, but it can already demonstrate the power and potential of the unified information framework. Its implementation in an in-house server can be scaled up by several magnitudes, being able to encompass data of billions of cells with a million features in the future.

Such a unified cell atlas not only has basic applications such as the annotation of query cells, but creates a brand-new scheme of characterizing biological entities in the atlas (anatomic units, cell types and genes) from all angles. A cell type is no longer only labeled by the up or down expression of a few marker genes, but is characterized by expression distributions of all relevant genes, participations in all organs and interactions with other cell types. Lineage and transient information will also be included when developmental or stimuli-response data are available. Similarly, a gene is characterized by its expression distributions in all cell types, subtypes and organs, its spatial and temporal patterns, and its relations with other genes. With the increase in data types and coverages, this will eventually increase our understanding of biological entities from sketchy ‘snapshots’ to quantitative ‘holographic portraits’ (Fig. 1).

The unified cell atlas can turn many biological experiments into computer codes. We use the typical biological experiment of cell sorting to study how a unified cell atlas can revolutionize biological experiments. Cell sorting is an experimental technique to select cells with desired properties from a human sample (*in vivo*) or cultured cells (*in vitro*). It is crucial for many scientific investigations. The number and types of features that can be used to select cells are restricted, and sorting cells from multiple *in vivo* samples is labor intensive and often infeasible.

A unified cell atlas can allow users to search for cells using combinational logic conditions virtually on all features, including expression of one or more genes, cell types or subtypes, source organs and metadata of the donors (sex, age, health status, etc.). An ultimate unified atlas will become a ‘virtual human body’ of cells in data space. Searching for cells with any desired properties can be implemented by computer codes exploring the virtual body. We call this scheme ‘in data’ cell sorting as it selects cells in the data body. Similar ideas have also been proposed in recent works such as Sfaira [11] and HCA Data Coordination Platform (DCP) (https://data.humancellatlas.org) for screening single-cell data using criteria on annotation labels or expression values, but they mainly focus on selections of datasets rather than cells.

We conducted experiments in the hECA to study this scheme based on the currently limited data coverage [10]. For example, we studied possible side effects of a chimeric antigen receptor T-cell (CAR-T) targeted cancer therapy. We used *in data* cell sorting for all cells that expressed the targeting protein across multiple organs from diverse donors. Analyzing effects on those cells and host organs enabled quick identification of the off-target organs (Fig. 1). These examples highlighted the power of the customized reuse of data in a unified atlas, although the current data are still limited.

There has been work that uses machine-learning methods to provide annotation for querying cells or to estimate the abundance of membership cell types in bulk data based on integration of single-cell reference data [7,12]. The unified assembly of massive scattered data into an atlas and the facility for agile construction of customized sub-atlases by in data sorting of wanted cells from the assembly can also provide better references for those methods.

The scientific community has exhibited huge enthusiasm in building cell atlases. The interests of most labs, however, focus on specific questions using single-cell technologies. The massive data they keep generating will form a great source for the assembly of cell atlases in a bottom-up manner, if the major informatics challenges can be well tackled. The ECA was a first attempt in this direction. It provided a prototype to showcase the promising strategy of a unified information framework for building a cell atlas from data of all sources.
